# *In vitro* and *in vivo* antiviral effects of CLEVir-X against porcine reproductive and respiratory syndrome virus

**DOI:** 10.1016/j.virusres.2024.199380

**Published:** 2024-04-27

**Authors:** Jeongmin Suh, Sehyeong Ham, Youngnam Kim, Sunghun Kim, Ahreum Cho, Hojin Moon, Chanhee Chae

**Affiliations:** a Department of Veterinary Pathology, College of Veterinary Medicine, Seoul National University, Seoul, South Korea; b CLEVir Lab, Strategy & Planning, CJ Cheiljedang BIO, Seoul, South Korea

**Keywords:** CLEVir-X, CLEVir-X PRRSV inhibition, CLEVir-X IMPDH inhibition, CLEVir-X mutagenesis, CLEVir-X antiviral action, CLEVir-X pig *in vivo* experiment

## Abstract

The aim of this study was to investigate the *in vitro* and *in vivo* antiviral effects of CLEVir-X, against porcine reproductive and respiratory syndrome virus (PRRSV). CLEVir-X is a nucleoside analogue and a dialdehyde form of xanthosine. CLEVir-X demonstrated antiviral action during the *in vitro* portion of this experiment with its inosine monophosphate dehydrogenase (IMPDH) inhibition against PRRSV. The anti-PRRSV effect of CLEVir-X was recovered through supplementation with guanosine. This suggests that PRRSV replication may be regulated through IMPDH and its guanosine biosynthetic pathway. CLEVir-X treatment in cultures resulted in mutation frequency increase of up to 7.8-fold within the viral genomes (*e.g.* ORF6) compared to their parallel, untreated cultures. The incorporation of CLEVir-X into the viral genome causes lethal mutagenesis and subsequent decrease in specific infectivity. During the *in vivo* antiviral experiment, 21-day-old pigs began oral administration of 5 mL of phosphate buffered saline containing CLEVir-X (with purity of 68 % and dosage of 40 mg/kg body weight). This treatment was provided twice daily at 9:00AM and 5:00PM for 14 days. Pigs were simultaneously intranasally inoculated with PRRSV at the beginning of CLEVir-X treatment (21 days of age). Several beneficial effects from the oral administration of CLEVir-X were observed including reduction of body temperature, alleviation of respiratory clinical signs, decreased PRRSV load in both blood and lung tissues, and mitigation of lung interstitial pneumonia lesions. The results of the present study demonstrated that CLEVir-X has mutagenic and nonmutagenic modes of antiviral action against PRRSV based on both *in vitro* and *in vivo* antiviral experiments.

## Highlights

• CLEVir-X inhibits PRRSV via disruption of IMPDH *in vitro*.
• CLEVir-X induced *in vitro* mutagenesis of PRRSV, reducing infectivity.
• Oral administration of CLEVir-X alleviated clinical signs, lung lesions and PRRSV load in experimentally infected pigs.

## 1 Introduction

Porcine reproductive and respiratory syndrome virus (PRRSV) is a non-segmented, positive sense and single stranded RNA animal virus which is a member of the family *Arteriviridae* and together with *Coronaviridae*, belongs to the order *Nidovirales* (Cavanagh, 1997). PRRSV is one of the costliest viral pathogens as it causes devastating disease to the global swine industry. PRRSV induces reproductive losses such as a substantial increase in late-term abortions and mummy incidences along with infertility. In pigs of all ages, it causes pneumonia with high morbidity and mortality and impacts production by slowing pig growth (Zimmerman et al., 2012).

PRRS modified-live virus (MLV) vaccines are widely used to combat the disease (Kimman et al., 2009; Murtaugh and Genzow, 2011; Renukaradhyaa et al., 2015). Although PRRS MLV vaccines confer complete protection against homologous viral strains, protection is limited against heterologous strains (Kimman et al., 2009; Murtaugh and Genzow, 2011; Renukaradhyaa et al., 2015). Efforts to achieve sustainable control of PRRS through vaccination have not yielded satisfactory results for multiple reasons. PRRSV consists of a multitude of genetically and antigenically diverse strains (Zimmerman et al., 2012). The extensive viral variability and inconsistent effectiveness of MLV vaccines pose formidable challenges in the development of consistently efficacious vaccines, especially under heterologous field conditions.

Consequently, application of antiviral agents is an alternative strategy to control PRRSV infection. In general, viruses heavily depend on the host cellular nucleotides, important constituents of RNA and DNA, to replicate and complete their life cycle. Inosine monophosphate dehydrogenase (IMPDH) catalyzes a rate-limiting first step essential enzyme for the de novo biosynthesis of guanine nucleotide (Hamilton et al., 2009; Zimmermann et al., 1996). Therefore, inhibition or disruption of IMPDH involved in nucleotides biosynthesis in host cells represented a validated target for potential antiviral strategy. Besides inhibition of IMPDH, antiviral agents such as nucleoside analogues act as an RNA virus mutagen (Crotty et al., 2000) where it was proposed that lethal mutagenesis is the critical mechanism of action of the antiviral agent (Crotty et al., 2001). The aim of the present study was to evaluate the *in vitro* and *in vivo* antiviral effect of the CLEVir-X (the dialdehyde form of xanthosine) on PRRSV replication.

## 2 Methods and materials

### 2.1 CLEVir-X (the dialdehyde form of xanthosine)

CLEVir-X, a dialdehyde form of xanthosine, was provided by CJ Cheiljedang (Seoul, Republic of Korea). The purity of CLEVir-X was 68 %.

### 2.2 Preparation of cells and viruses for *in vitro* test

RPMI 1640 medium (Gibco, Gaithersburg, MD) was supplemented with 5 % fetal bovine serum (FBS; HyClone, Logan, UT), and Penicillin-Streptomycin solutions (100 ×; Gibco) for use with MARC-145 cells. The cells were propagated at 37 °C in a CO_2_ incubator set to 5 % humidity. MARC-145 cells were used to propagate PRRSV (SNUVR090851 strain, Lineage 1, GenBank no**.** JN315685).

### 2.3 Cytotoxicity assay of CLEVir-X

Cytotoxicity of CLEVir-X was evaluated on MARC-145 cells with a commercially available water-soluble tetrazolium salt-1 (WST-1) assay (EZ-Cytox; DoGenBio, Seoul, Korea) in accordance with the manufacturer's protocol. Briefly, 96-well tissue culture plates were planted with MARC-145 cells at density of 2 × 10^4^ cells/well and incubated for 1 day prior to use in the cytotoxicity study. Then, the cells were treated with various concentrations (0 to 10,000 μM) of CLEVir-X for 24 h. After 24 h, supernatant was removed from all wells of the plate, and 100 μL of WST-1 substrate solution was added. Substrate solution was developed for 1 h. Solution absorbance was then measured using an Epoch Microplate spectrophotometer (BioTeK Instrumentals, Inc., Winooski, VT) at a UV wavelength of 450 nm. All WST-1 assays were performed in triplicate.

### 2.4 Antiviral activity assay

An indirect immunofluorescence assay (IFA) was used to analyze the antiviral activity of CLEVir-X. MARC-145 cells were planted 1-day prior to use in 96-well tissue culture plates. The 1-day-old monolayers were then either mock infected or infected with PRRSV (SNUVR090851 strain) at 1 × 10^2^ TCID_50_/mL in the presence or absence of CLEVir-X, and the plates were returned to the incubator for 72 h to allow the virus to propagate. Then, the cells were fixed and permeabilized with a 1:1 ratio of methanol:acetone for 30 min at −20 °C. Following virus fixation, all wells of the 96-wells plates were washed with PBS three times and blocked with PBS containing 1 % bovine serum albumin (BSA) for 30 min at room temperature. Post blocking, monoclonal antibody SDOW17 (1;1000 in PBS containing 0.1 % Tween 20, Rural Technologies, Brookings, SD) was used as the primary antibody, incubated on the plate for 2 h at room temperature. Post-incubation, all wells of the plates were washed three times with PBS prior to secondary antibody addition. A goat-anti-mouse detector conjugated with Alexa Fluor 488 (Invitrogen, Carlsbad, CA) was incubated on the plates for 1 h at room temperature. Virus infectivity and the efficacy of CLEVir-X were calculated by first counterstaining the cell nuclei. 4′, 6-diamidino-2-phenylindole (DAPI; Sigma, St. Louis, MO) was added to the plates and incubated for 10 min. Excess stain was removed and background reduced by washing the plates three times with PBS prior to reading. The cell staining was visualized using a fluorescent Olympus BX43 (Olympus, Center Valley, PA) and infectivity was calculated by counting the number of stained and unstained cells. All assays were performed in triplicate.

### 2.5 Exogenous guanosine supplementation analysis

PRRSV-infected MARC-145 cells were prepared and incubated for 72 h, as described above, and treated either with or without a 100 μM concentration of guanosine in the presence or absence of 200 μM CLEVir-X. As a positive control, Mycophenolic acid (MPA; Sigma), an IMPDH-specific inhibitor, served as a positive control for the assay, and was also subjected to guanosine. The assay fixed virus-infected cells to the plates and was analyzed as described above through IFA.

### 2.6 Confirmation of mechanism of CLEVir-X to generate progeny incomplete in infectivity

MARC-145 cells that reached 100 % cell confluency were infected with PRRSV in both the presence or absence of an 80 % effective concentration (EC_80_, =80 μM) of CLEVir-X for 72 h. Progeny virus particles were titrated by real-time PCR using ORF5-specific primer sets (PRRSV ORF5 forward, 5′-AAACCAGTCCAGAGGCAAGG-3′; PRRSV ORF5 reverse, 5′-GCAAACTAAACTCCACAGTGTAA-3′). This was done to validate if there were any changes in infectivity by normalizing the progeny numbers for subsequent infection. Control progeny virus and the CLEVir-treated progeny virus were cultured in equal particle numbers onto new sets of MARC-145 cells post-titration. At 72 h post infection (hpi), the virus-infected cells were fixed and subjected to IFA to evaluate the presence of PRRSV infection as described above.

### 2.7 Measurement of viral mutation frequency

Viral mutation frequency by CLEVir-X, in the structural protein encoding regions (ORF2 to ORF6) of PRRSV genes, was evaluated as follows: MARC-145 cells were infected with PRRSV or infected with PRRSV under CLEVir-X treatment (EC_80_). At 72 hpi, a total RNA extraction kit (RNA-spin™, iNtRON Biotechnology, Seongnam, Korea) was used to extract total RNA from the incubated plates. RT-PCR was performed to amplify the region encoding the structural proteins of PRRSV using gene-specific primer sets (PRRSV structural protein forward, 5′- GACCTCGCGGTCACCCCTTATGATTACGGC-3′; PRRSV structural protein reverse, 5′-ATCTGACAGGGTGCAAGTCCCAGCGCCTTG-3′). Product was selected after 10 independent PCR cycles were performed and analyzed for pattern and frequency of mutations in the region.

### 2.8 *In vitro* viral RNA-dependent RNA polymerase inhibition assay

A SARS-CoV-2 RNA Polymerase Assay Kit (ProFoldin, Hudson, MA) was used in accordance with the manufacturer's protocol to examine *in vitro* viral RNA-dependent RNA polymerase (RdRp) inhibition. Briefly, recombinant RNA polymerase was incubated with template (as a single-stranded polyribonucleotide), NTPs and CLEVir-X at 25, 50, 100, 500 and 1000 μM concentrations. The mixture was incubated for 120 min at 37 °C, at which point the reaction was stopped with an addition of 130 μL of 1 × fluorescence dye. A fluorescence spectrometer set to read fluorescence signal at extension/emission = 485/535 nm wavelength was used within 5 min post-stop to observe the inhibitory efficacy of CLEVir-X on PRRSV replication.

### 2.9 *In vivo* antiviral effect against PRRSV in pigs

Twenty-four clinically healthy weaned piglets were purchased from a commercial PRRSV free farm at 18 days of age. Piglets screened as seronegative for PCV2 (INgezim CIRCO IgG, Ingenasa, Madrid, Spain), *Mycoplasma hyopneumoniae* (*M. hyo* Ab test, IDEXX Laboratories Inc. Westbrook, ME), and PRRSV (HerdChek PRRS X3 Ab test, IDEXX Laboratories Inc.).

The experimental study contained three randomly divided groups (*n* = 8 pigs per group). Treatment began on pigs in the CLEVir-X+PRRSV group at 21 days of age where they were orally administered 5 mL of phosphate buffered saline (PBS, 0.01 M, pH 7.2) containing a crude dialdehyde form of xanthosine (purity 68 %, 40 mg/kg body weight) twice daily (9:00AM and 5:00PM) for 14 days. Simultaneously at the time of the CLEVir-X treatment (21 days of age), 0 days post challenge (dpc), pigs in the CLEVir-X+PRRSV and PRRSV groups were each inoculated with 3 mL of PRRSV (SNUVR090851 strain, Lineage 1, GenBank no. JN315685) tissue culture fluid containing 10^5^ 50 % tissue culture infective dose (TCID_50_)/mL (fourth passage in MARC-145 cells). The challenge PRRSV strain was isolated in 2010 from lung samples of newly weaned pigs from a 1000-sow herd located in the Chungcheong Province (Han et al., 2013). Pigs in the control group were administered 3 mL of PBS by the same route as the challenge. At 14 dpc (35 days of age), pigs were sedated by an intravenous injection of sodium pentobarbital and then euthanized by electrocution as previously described (Beaver et al., 2001). Tissues were collected from each pig at necropsy. All of the methods were previously approved by the Seoul National University Institutional Animal Care and Use Committee (approval no. SNU-230718-4).

### 2.10 Clinical observations

The pigs were monitored daily for physical conditions and scored weekly for clinical respiratory disease severity using scores ranging from 0 (normal) to 6 (severe dyspnea and abdominal breathing) (Halbur et al., 1995). Observers were blinded to treatment status. Rectal temperatures were measured and recorded at 0, 4, 7, 11, and 14 dpc at the same time by the same personnel.

### 2.11 Body weight and average daily weight gain

Pig weight was measured at 0 (21 days of age) and 14 (35 days of age) dpc throughout the study. An average daily weight gain (ADWG = grams/pig/day) was calculated over time point between 0 and 14 dpc. The difference between the initial final weights were divided at each of these three time points by the number of days in the corresponding period to calculate ADWG. All data were obtained in a blinded manner.

### 2.12 Quantification of PRRSV cDNA in lung of PRRSV-infected pigs

Blood samples were collected at 0, 7, 11, and 14 dpc. RNA was extracted from serum samples to quantify PRRSV genomic cDNA copy numbers, as previously described (Wasilk et al., 2004).

### 2.13 Histopathological assessment

Microscopic lung lesions were examined and scored based on a scale of 0 (normal) to 4 (severe diffuse) based on the severity of interstitial pneumonia as previously described (Halbur et al., 1995).

### 2.14 Statistical analysis

For statistical processing, real-time PCR data were converted into decimal logarithmic values. Normal distribution was determined with the Shapiro-Wilk test on these data. Whether or not the groups had statistically significant differences between them at various timepoints was then determined by performing one-way ANOVA. For further evaluation, post-hoc test for pairwise comparison with Tukey's adjustment was conducted with a statistical significance result from one-way ANOVA test. Kruskal-Wallis test was additionally performed only in cases where the normality assumption was not met. Results which showed statistical significance from the Kruskal-Wallis test were further evaluated with the Mann-Whitney test to compare the differences among the groups. Results were reported in p-values and values of *P* < 0.05 were considered significant.

## 3 Results

### 3.1 Cytotoxicity assay of CLEVir-X

**Fig. 1:**
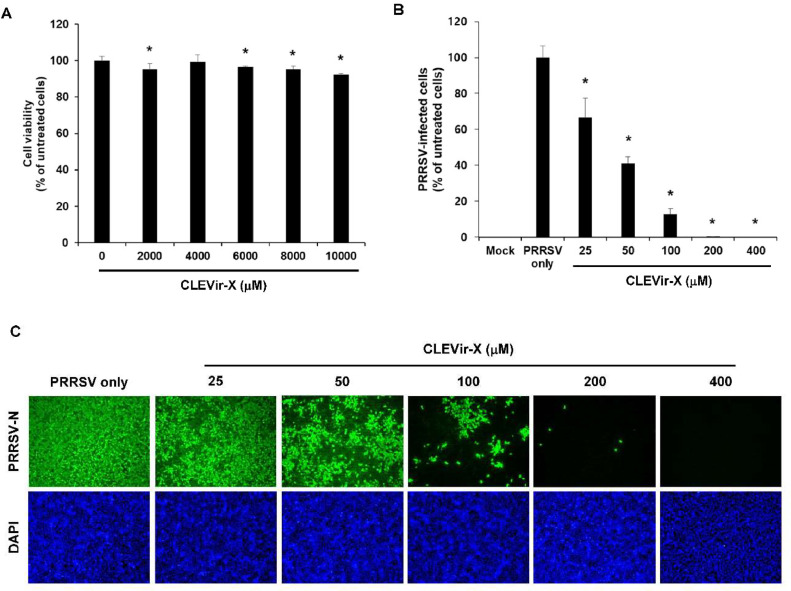
**Cytotoxicity and *in vitro* efficacy of CLEVir-X.** A) Viability of MARC-145 cells upon treatment with CLEVir-X at various concentrations, B) Antiviral effect of CLEVir-X on the replication of PRRSV. PRRSV infected MARC-145 cells were simultaneously treated with CLEVir-X at indicated concentrations (25 to 400 μM), and the infectivity and EC_50_ was calculated, and C) Immunostaining of anti-PRRSV N protein and DAPI staining. Data were representative of the mean of three independent experiments and error bars represent standard deviations (*P = 0.001–0.05).

CLEVir-X was determined as cytotoxic before assessing its antiviral activities. MARC-145 cells were treated for 24 h with different concentration of the CLEVir-X ranging from 0 to 10,000 μM. CLEVir-X failed to distinctly affect cell viability on MARC-145 cells at its highest concentration (10,000 μM) post-24 h treatment (Fig. 1A).

### 3.2 Antiviral activity of CLEVir-X

An IFA assay was implemented to observe cell morphology through cytopathic effect induced by PRRSV propagation. Dose-dependent inhibition of PRRSV was observed by CLEVir-X treatment (Figs. 1B and C), where approximately 40 μM of CLEVir-X was disclosed as 50 % of the effective concentration in PRRSV replication inhibition.

### 3.3 Reduction of PRRSV replication through inhibition of IMPDH function

**Fig. 2:**
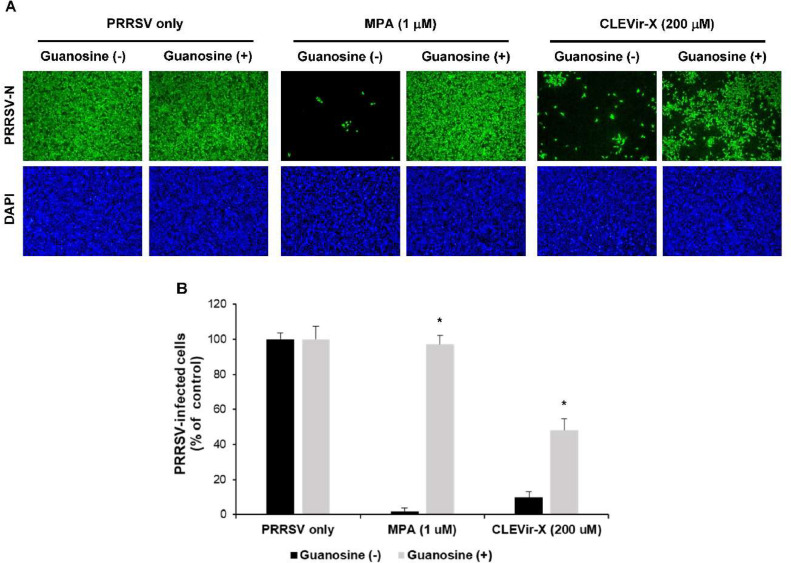
Effect of supplementation of guanosine in **CLEVir-X or MPA on PRRSV infection.** A) MARC-145 cells were treated with CLEVir-X (200 μM) or MPA (1 μM) with or without 100 μM of guanosine. PRRSV-infected cells were fixed and subjected to immunofluorescence assay, and B) Infectivity was measured by quantifying the number of cells expressing N proteins by immunofluorescence assay. Data were representative of the mean values from three independent experiments and error bars represent standard deviations (*P < 0.001).

To further examine the relevance of the guanosine nucleotide synthetic pathway in the anti-PRRSV activity of CLEVir-X, supplementation with guanosine clearly recovered the PRRSV infectivity. This suggests that IMPDH and its guanosine synthesis pathway are targets for the observed anti-PRRSV activity of CLEVir-X. As a positive control, MPA, an IMPDH specific inhibitor, was tested and compared with CLEVir-X to verify precise inhibitory function against IMPDH (Fig. 2A and B). Thus, IMPDH may be crucial for the efficient replication of PRRSV.

### 3.4 Mutagenic effects of CLEVir-X on PRRSV

**Fig. 3:**
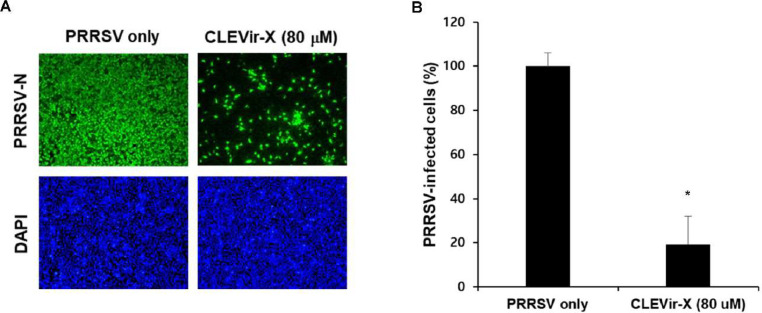
**Confirmation of infectivity of progeny virus.** A) MARC-145 cells were infected with PRRSV in the presence or absence of 80 μM of CLEVir-X, the concentration of which provides a minimum replication of PRRSV. Subsequently, the newly generated progenies were re-infected in MARC-145 cells and evaluated by immunofluorescence, B) Infectivity of progencies was measured by quantifying the number of cells expressing N proteins by immunofluorescence assay. Data were expressed as the mean values from three independent experiments and error bars represent standard deviations (*P < 0.001).

Clonal sequencing was used on a single passage of PRRSV-infected MARC-145 cells to evaluate whether CLEVir-X might act as a mutagen of PRRSV. The mutation frequency assay was performed by passing PRRSV on cells in the presence of CLEVir-X at 80 % of the effective concentration (EC_80_, = 80 μM). We then amplified and analyzed cDNA of 2827-bases on structural protein encoding regions (ORF2 to ORF6) from RNA in the culture supernatant. These amplified cDNAs, which included sequences from both mutated and nonmutated progenies, were sequenced. As shown in Table 1, statistically significant increase in the overall mutation frequency for viruses passaged in 80 μM CLEVir-X (*P* = 0.013 by chi-square test) was observed in the direction of losing infectivity in progenies (Fig. 3A and B).

**Table 1:** Summary of mutation noted in sequence analysis of wild-type and CLEVir-X treated PRRSV structural protein (ORF2-ORF6) gene.

Groups	Identity (%)	ORF	Mutation Category		
			Transition	Transversion	Total
PRRSV	99.03	ORF2	4.41	2.46	6.87
		ORF3	1.70	1.70	3.40
		ORF4	2.98	1.12	4.10
		ORF5	6.80	6.30	13.10
		ORF6	1.90	4.76	6.67
CLEVir-X +PRRSV	98.06	ORF2	3.63	7.39	11.02
		ORF3	1.31	3.66	4.97
		ORF4	1.12	2.52	3.54
		ORF5	8.13	15.59	23.71
		ORF6	18.10	34.48	52.57

Lethal mutagenesis is recognized by causing a reduction in the specific infectivity of a viral population (Fig. 3A). As mutations induced by the CLEVir-X accumulate in progeny genomes, fewer of the corresponding virions maintain infectivity (Fig. 3B). The specific infectivity of CLEVir-X-treated viral populations was calculated relative to mock-treated control samples based on titer. Virus that was treated with 80 μM CLEVir-X experienced an approximate 5-fold reduction in specific infectivity. Mutagenic action was attributed to CLEVir-X if the nucleotide sequencing of the structural protein encoding regions (ORF2 to ORF6) was obtained from the mutant spectrum of PRRSV after CLEVir-X treatment.

**Fig. 4:**
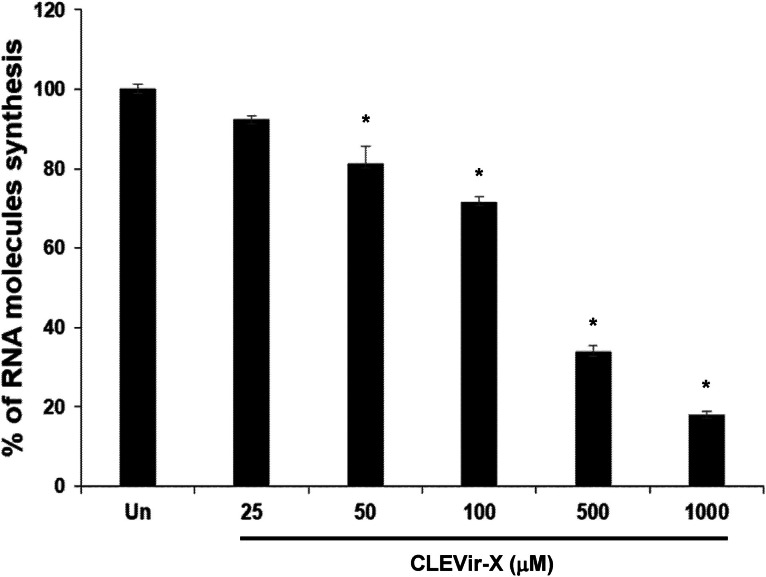
**Inhibitory effect of CLEVir-X on viral RNA-dependent RNA polymerase (RdRp).** Replicational function of recombinant RdRp (SARS-CoV-2) enzyme was inhibited by CLEVir-X under dose-dependent manner. The bars indicate% of synthesized RNAs and the% was relatively calculated based on untreated. Results are expressed as% of control without CLEVir-X and error bars represent standard deviations (*P < 0.001).

CLEVir-X was tested with SARS-CoV-2 RNA Polymerase Assay Kit to determine its inhibition of viral RdRp during RNA replication. According to the collected data, CLEVir-X efficiently capped SARS-CoV-2 RdRp activity under a dose-dependent manner (Fig. 4). Under these finding along with the process of mutagenic function, CLEVir-X should have various means in an antiviral mode to show a certain effect.

### 3.5 Clinical observations

**Fig. 5:**
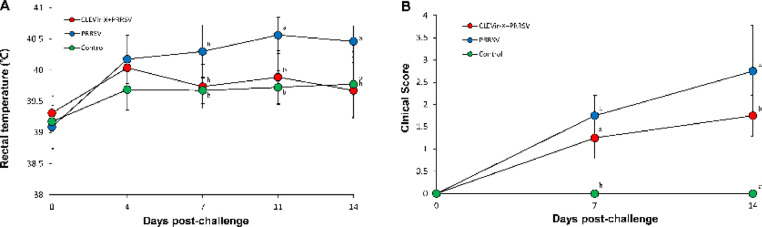
**Clinical information.** A) Body temperature and B) Respiratory clinical score. In CLEVir-X+PRRSV group, pigs were orally administered with CLEVir-X and were intranasally inoculated with PRRSV. In PRRSV group, pigs were intranasally inoculated with PRRSV. In control group, pigs were administered phosphate buffered saline. Variation is expressed as the standard deviation. Significant differences (*P* < 0.05) were observed within a sampling point mean. Different superscripts (a, b, and c) indicate statistical differences among 3 groups.

Prior to challenge, pig rectal temperatures were within the normal range (38.6–39.7 °C) for all the three groups. By 7, 11, and 14 dpc, rectal temperatures of the CLEVir-X+PRRSV and control groups were significantly (*P* < 0.05) lower than those of the PRRSV group (Fig. 5A). Pigs in the CLEVir-X+PRRSV and control groups had significantly (*P* < 0.05) lower respiratory clinical scores at 14 dpc than those in the PRRSV groups. Pigs in the control group had significantly lower (*P* < 0.05) respiratory clinical scores than those in the CLEVir-X+PRRSV group at 7 and 14 dpc (Fig. 5B).

### 3.6 Body weight and average daily weight gain

Difference in mean body weight was not observed among 3 groups between vaccinated and at the beginning (21 days of age) and the end (35 days of age) of study. Pigs in the PRRSV group had significantly (*P* < 0.05) lower ADWG at 35 days of age compared to the control group (Table 2).

**Table 2:** Body weight, average daily weight gain, and microscopic lung lesions in 3 groups.

	Age (days)	Groups		
		CLEVir-X+PRRSV	PRRSV	Control
Body weight	21	4.88 ± 0.89	4.64 ± 0.90	4.53 ± 1.26
	35	7.83 ± 1.48	6.26 ± 1.62	8.01 ± 2.37
ADWG	21–35	210.71 ± 61.92^ab^	116.07 ± 66.98^a^	248.21 ± 99.62^b^
Lung lesion score	35	2.25 ± 0.19^a^	2.8 ± 0.28^b^	0 ± 0^c^

Different superscript letters (a, b, and c) mean statistically significant differences (*P* < 0.05).

### 3.7 Quantification of prrsv rna

**Fig. 6:**
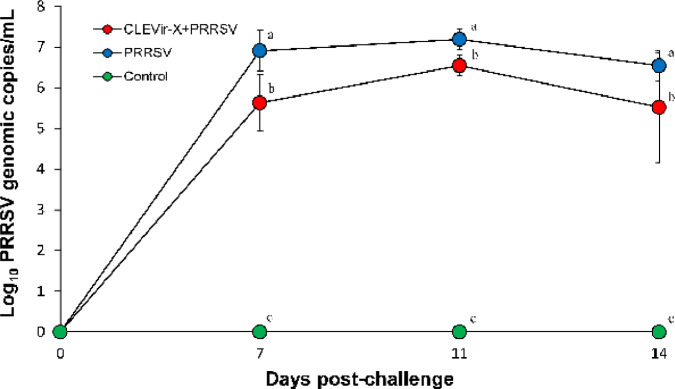
**Mean values of the genomic copy numbers of PRRSV cDNA in serum**. In CLEVir-X+PRRSV group, pigs were orally administered with CLEVir-X and were intranasally inoculated with PRRSV. In PRRSV group, pigs were intranasally inoculated with PRRSV. In control group, pigs were administered phosphate buffered saline. Variation is expressed as the standard deviation. Significant differences (*P* < 0.05) were observed within a sampling point mean. Different superscripts (a, b, and c) indicate statistical differences among 3 groups.

The number of PRRSV genomic copies in serum samples of the CLEVir-X+PRRSV group were significantly (*P* < 0.05) lower than those of the PRRSV group on 7, 11, and 14 dpc (Fig. 6). The number of PRRSV genomic copies in lung samples of the CLEVir-X+PRRSV group was significantly (*P* < 0.05) lower than that of the PRRSV group on 14 dpc. PRRSV was not detected in the control group throughout the entire study.

### 3.8 Pathology

**Fig. 7:**
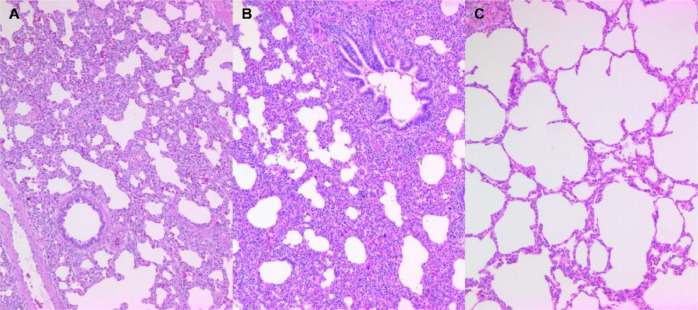
**Histopathology.** A) Mild interstitial pneumonia was observed in pigs orally administered with CLEVir-X and intranasally inoculated with PRRSV, B) Severe interstitial pneumonia was observed in pigs were intranasally inoculated with PRRSV, C) Normal lungs from control pig.

The lung lesion score of the CLEVir-X+PRRSV group (Fig. 7A) was significantly (*P* < 0.05) lower than that of the PRRSV group (Fig. 7B) on 14 dpc. Lung lesions were not detected in the control group (Fig. 7C) throughout the entire study (Table 2).

## 4 Discussion

CLEVir-X demonstrates antiviral action via IMPDH inhibition against PRRSV. IMPDH catalyzes the conversion of inosine 5′-monophosphate into xanthosine 5′-monophosphate, biosynthesizing the guanosine nucleotide which is an intermediate in the de novo synthesis of guanosine (Feld and Hoofnagle, 2005). RNA synthesis requires IMPDH regulation of the intracellular GTP pools, allotting for the antiviral mechanisms of inhibitor compounds against both DNA and RNA viruses (Sidwell et al., 1972). Intracellular GTP was depleted in the present study by CLEVir-X disrupting the enzymatic activity of IMPDH. To test this antiviral mechanism theory in relation to PRRSV, guanosine was added to infected cells that were treated with CLEVir-X. An anti-PRRSV effect was not observed in cells treated solely with guanosine, but it did attenuate the anti-PRRSV effects of CLEVir-X significantly. These findings support the suggestion that nucleotide depletion by CLEVir-X is an important mechanism in controlling PRRSV infection.

The mutagenic antiviral action that CLEVir-X provides against PRRSV is also determined through mutation frequency and the specific infectivity of progeny assessment. CLEVir-X also has an antiviral activity by increasing frequency of mutation on the genes encoding structural proteins of PRRSV which resulted in loss of infectivity of the progenies. ORF5 and ORF6 are major components to generate the viral envelope which is critical to further infection of neighbors. Therefore, induction of dysfunctional envelope structure by mutagenic function of CLEVir-X was as significant as the inhibition of IMPDH to explain antiviral effect of CLEVir-X. All of these results provide clinically useful information. To-date, there is a lack of efficacious commercially available PRRS vaccines. As an RNA virus, antiviral treatments are difficult to implement due to the highly variable quasi-species dynamic of PRRSV (Domingo et al., 2012). This quasi-species dynamic combined with rapid mutation rate provides PRRSV with its viral persistence and infection-caused disease (Goldberg et al., 2003; Montaner-Tarbes et al., 2019; Wills et al., 1997). CLEVir-X and its lethal mutagenesis observed here, may decrease the infectivity of PRRSV progeny and shorten its viral persistence. A similar antiviral mutagenesis mechanism (nucleoside analogue ribavirin-mediated) has been reported in polio virus and foot and mouth disease (Airaksinen et al., 2003; Crotty et al., 2000). This similarity between the viruses is relevant to our data as CLEVir-X inhibits IMPDH, while lethal mutagenesis is considered the critical anti-viral action of CLEVir-X against PRRSV replication.

Although IMPDH inhibitors have been noted to show antiviral activity against some viruses (Sidwell et al., 1972), many of them have failed to exhibit promising antiviral effects in animal models. However, in the present study, the antiviral effects of CLEVir-X were also observed in the animal experiment, where pigs treated with CLEVir-X exhibited lower amounts of PRRSV blood and lung viral loads and lower microscopic interstitial pneumonia severity, when compared with the control group.

Oral administration of CLEVir-X can protect pigs against an intranasal PRRSV challenge. The potential antiviral effect that CLEVir-X had on blood and lung viral loads was perhaps the most interesting clinical outcome of this study. PRRSV viremia plays a key role in the development of respiratory disease and virus distribution throughout the body (Cheon and Chae, 1999; Han et al., 2013; Lager et al., 2014). Therefore, PRRSV viremia is considered the crucial index in the evaluation of the antiviral effect of CLEVir-X on PRRSV. Treatment with CLEVir-X statistically reduced the amount of PRRSV viral load in the blood and the lungs compared to untreated control pigs. The reduction of blood and lung viral load alleviated respiratory clinical signs through decrease in the severity of interstitial pneumonia and reduction in body temperature. Due to the small sample size of pigs used per group, and the short observation window post-PRRSV challenge, significant difference in body weight and ADWG between the CLEVir-X treated and untreated groups was not found. Nevertheless, numerical differences may be also clinically meaningful information. Pig rearing conditions in overly-regulated experimental conditions are different from field conditions, where pigs continue to expose and re-expose themselves to field PRRSV by horizontal and vertical transmission, which exacerbates the disease.

Although IMPDH inhibitors have been noted to show antiviral activity against some viruses (Sidwell et al., 1972), many of them have failed to exhibit promising antiviral effects in animal models. However, this study successfully demonstrated the inhibitory effect that CLEVir-X has on PRRSV replication through both *in vitro* and *in vivo* methodology. Pigs treated with CLEVir-X exhibited lower amounts of PRRSV blood and lung viral loads and lower microscopic interstitial pneumonia severity, when compared with the control group. The development of immunity against PRRSV (unlike other viral diseases) requires a minimum of 42 days in MLV-vaccinated pigs (Kimman et al., 2009; Murtaugh and Genzow, 2011). Furthermore, MLV vaccines confer variable degrees of protection against heterologous strains (Kimman et al., 2009; Murtaugh and Genzow, 2011; Renukaradhyaa et al., 2015). Antiviral agents have an advantage here, as they produce an immediate effect while remaining unaffected by viral antigenicity and genetic variability. As a result, they minimize the spread of infection and reduce damage to pigs within herds where wild-type PRRSV circulates. The results of the present study demonstrated that CLEVir-X has mutagenic and nonmutagenic modes of antiviral action against PRRSV based on both *in vitro* and *in vivo* antiviral experimentation.

## 5 Conclusions

CLEVir-X demonstrated *in vitro* antiviral action with its inosine monophosphate dehydrogenase (IMPDH) inhibition against PRRSV. The anti-PRRSV effect of CLEVir-X was recovered through supplementation with guanosine. During the *in vivo* antiviral experiment, beneficial effects from the oral administration of CLEVir-X were observed including reduction of body temperature, alleviation of respiratory clinical signs, decreased PRRSV load in both blood and lung tissues, and mitigation of lung interstitial pneumonia lesions. The results of the present study demonstrated that CLEVir-X has mutagenic and nonmutagenic modes of antiviral action against PRRSV based on both *in vitro* and *in vivo* antiviral experiments.

## CRediT authorship contribution statement

**Jeongmin Suh:** Methodology, Data curation, Writing – original draft, Investigation. **Sehyeong Ham:** Methodology, Data curation, Investigation. **Youngnam Kim:** Resources, Data curation, Methodology. **Sunghun Kim:** Resources, Project administration, Funding acquisition. **Ahreum Cho:** Resources, Software. **Hojin Moon:** Validation, Data curation. **Chanhee Chae:** Conceptualization, Supervision, Writing – review & editing, Writing – original draft, Project administration.

## Declaration of competing interest

The authors declare that they have no known competing financial interests or personal relationships that could have appeared to influence the work reported in this paper.

## Data Availability

Data will be made available on request.
